# Premature Bioprosthetic Mitral Valve Dysfunction Due to Flail Leaflet Treated With Transcatheter Mitral Valve Replacement; a Case Report

**DOI:** 10.7759/cureus.30814

**Published:** 2022-10-28

**Authors:** Carlos Munoz, Carla Valencia, Christopher Scoma, Joel Fernandez

**Affiliations:** 1 Internal Medicine, California Institute of Behavioral Neurosciences & Psychology, Fairfield, USA; 2 Cardiology, University of South Florida, Morsani College of Medicine, Tampa, USA; 3 Cardiology, University of South Florida, Tampa, USA

**Keywords:** interventional cardiology, echocardiography - heart failure - valvular heart disease, bioprosthetic valve dysfunction, transcatheter mitral valve-in-valve replacement, heart failure

## Abstract

Early bioprosthetic mitral valve failure is uncommon, but cases can present with symptomatic heart failure and require careful attention and evaluation. Transcatheter valve replacement is a minimally invasive treatment for mitral valve dysfunction that can have a considerable impact, particularly for those who are at high surgical risk or have a history of bioprosthetic valve failure. We present a rare case of significant mitral regurgitation due to the unexpected premature failure of a bioprosthetic valve that was implanted three years prior. This patient was treated with transcatheter mitral valve replacement with the implantation of an Edwards SAPIEN Ultra (Edwards Lifesciences) valve.

## Introduction

Mitral regurgitation (MR) is the most common valvular disease, affecting over 10% of patients over 75 years old. Regurgitant flow can occur due to several factors owing to the complex geometry of the mitral apparatus, which encompasses a saddle-shaped annulus, two non-uniform leaflets, and a sophisticated subvalvular apparatus. Furthermore, the valve is dynamic, changing in size and shape throughout the cardiac cycle, leading to a wide range of potential etiologies for dysfunction [1, 2, 3].

Treatment of severe mitral valve dysfunction has historically evolved through surgical valve repair or replacement; both remain the gold standard. Replacement with mechanical valves provides durable results, whereas replacement with bioprostheses tends to fail earlier [4]. However, the failure of the bioprosthetic valve within five years of implantation is rare, and there is a paucity of literature on this topic.

Newer technologies have allowed the use of transcatheter repair or replacement as a less invasive treatment for mitral valve dysfunction, especially for individuals with prohibitive surgical risk. The viability of the transcatheter mitral valve replacement (TMVR) inside a pre-existing bioprosthetic valve has been well-established, with a significant decrease in MR severity and improvement in symptoms [1, 3, 4]. 

We present a case of a symptomatic premature failure of a 31mm St. Jude Medical (Abbott Vascular) mitral valve bioprosthesis replaced by TMVR with a 29mm Edwards SAPIENS Ultra (Edwards Lifesciences) valve.

## Case presentation

**Figure 1:**
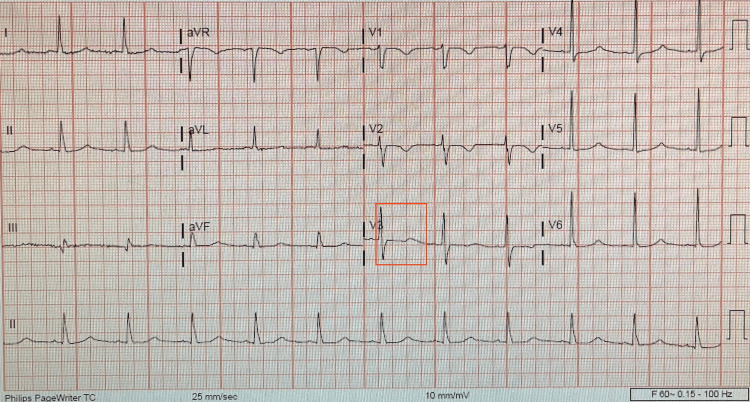
Electrocardiogram of the patient at the time of admission with prolonged QT interval (red box).

A 64-year-old female with a past medical history of mitral valve prolapse with severe mitral valve regurgitation and bioprosthetic valve placement in 2019 (31 mm St. Jude Medical valve), hypertension, atrial fibrillation, iron deficiency anemia, and hyperlipidemia presented to the emergency room with multiple episodes of dizziness, dyspnea on exertion, and pre-syncope for 10 days. The patient's vital signs on arrival included a blood pressure of 80/32 mmHg, heart rate of 112 beats per minute, and oxygen saturation of 95% on room air. The patient appeared anxious and pale and had a grade VI systolic murmur that was best appreciated on the lower left sternal border and with the presence of a palpable thrill. Initial laboratory evaluation revealed a chronically low hemoglobin level (10.5 mg/dL) and a normal white blood cell count. Her electrolytes and renal function tests were within normal limits. A chest x-ray demonstrated mild pulmonary venous congestion. An electrocardiogram demonstrated an atrial prolonged QT interval without significant ST segment or T-wave abnormalities (Figure 1). She was admitted to the coronary care unit for further management and evaluation with a diagnosis of congestive heart failure.

**Figure 2:**
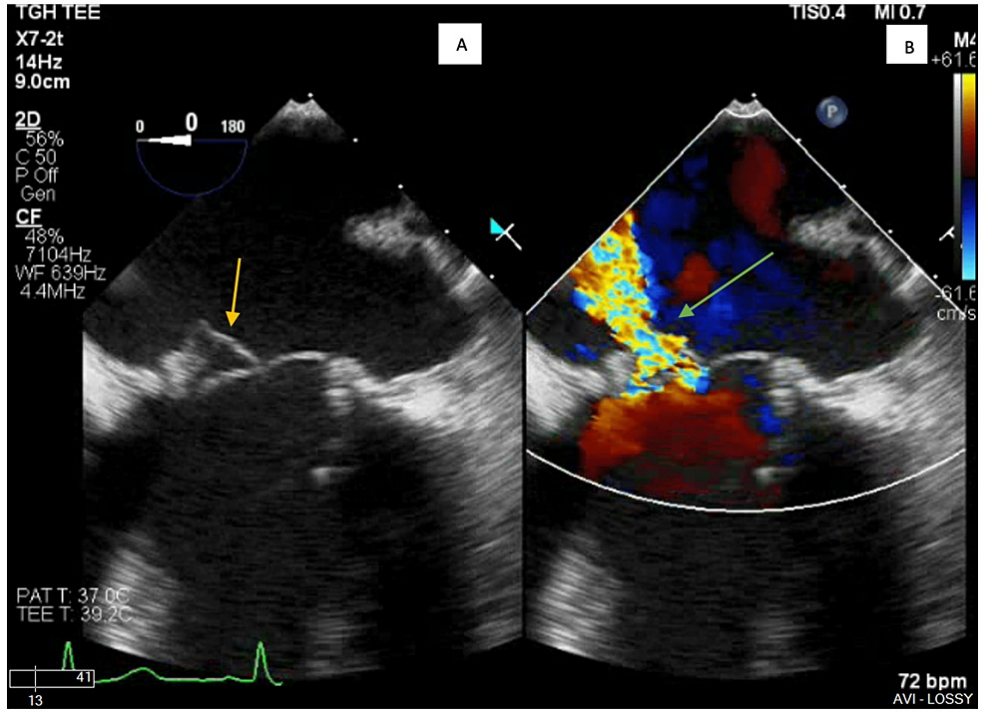
A) TEE of the bioprosthetic MV with flail anterior prosthetic leaflet (yellow arrow). B) Severe eccentric mitral regurgitation (green arrow). TEE: Transesophageal echocardiogram; MV: Mitral valve

**Figure 3:**
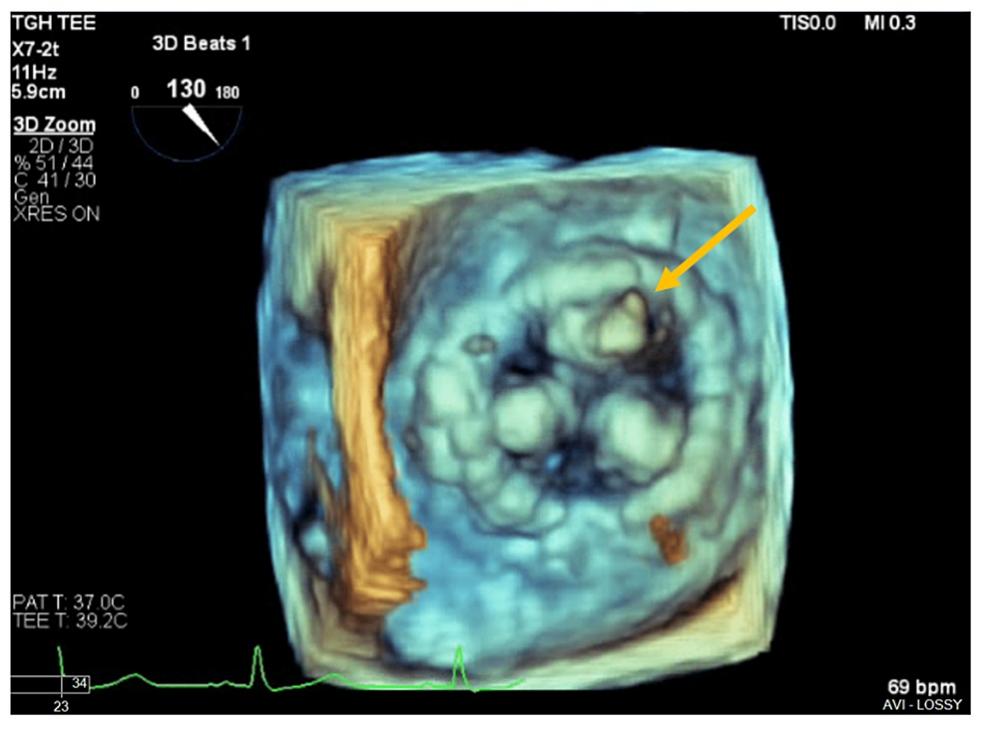
Three-dimensional TEE of the bioprosthetic mitral valve (surgeon’s view), without evidence of vegetation, thrombus, or pannus. The flail anterior leaflet is visible (yellow arrow). TEE: Transesophageal echocardiogram

Since murmurs and thrills were detected in the heart, prosthetic mitral valve dysfunction was suspected, and a transesophageal echocardiogram (TEE) was ordered for subsequent evaluation. The TEE revealed a left ventricular ejection fraction of 60-65% and an avulsion of the anterior bioprosthetic leaflet with a resultant large eccentric jet of severe mitral regurgitation; there was no evidence of bioprosthetic valve thrombosis, pannus, or vegetation (Figure 1, 2, 3).

**Figure 4:**
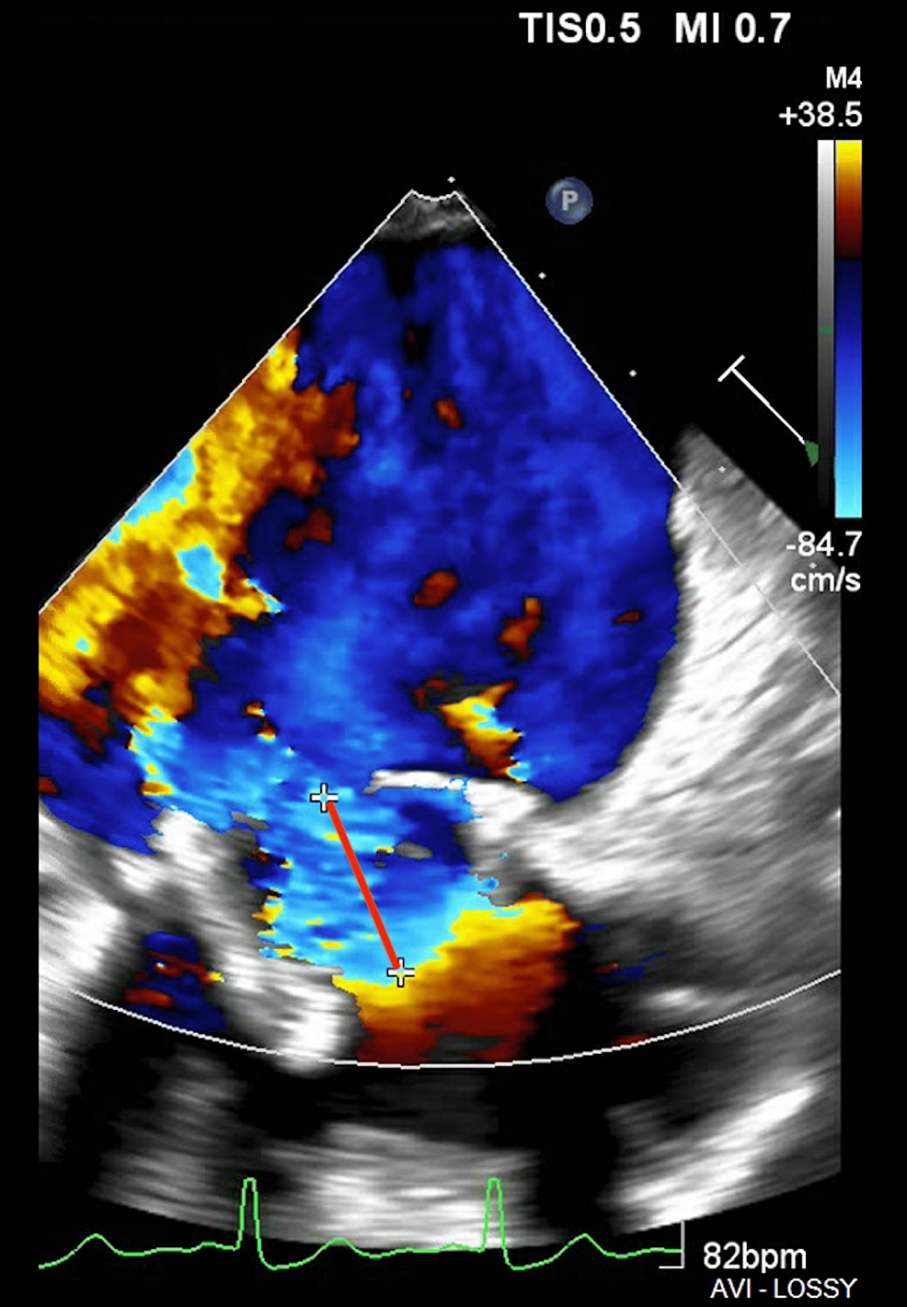
Color Doppler of the severe mitral regurgitation demonstrating a proximal isovelocity surface area (PISA) of 1.2cm (red line).

**Figure 5:**
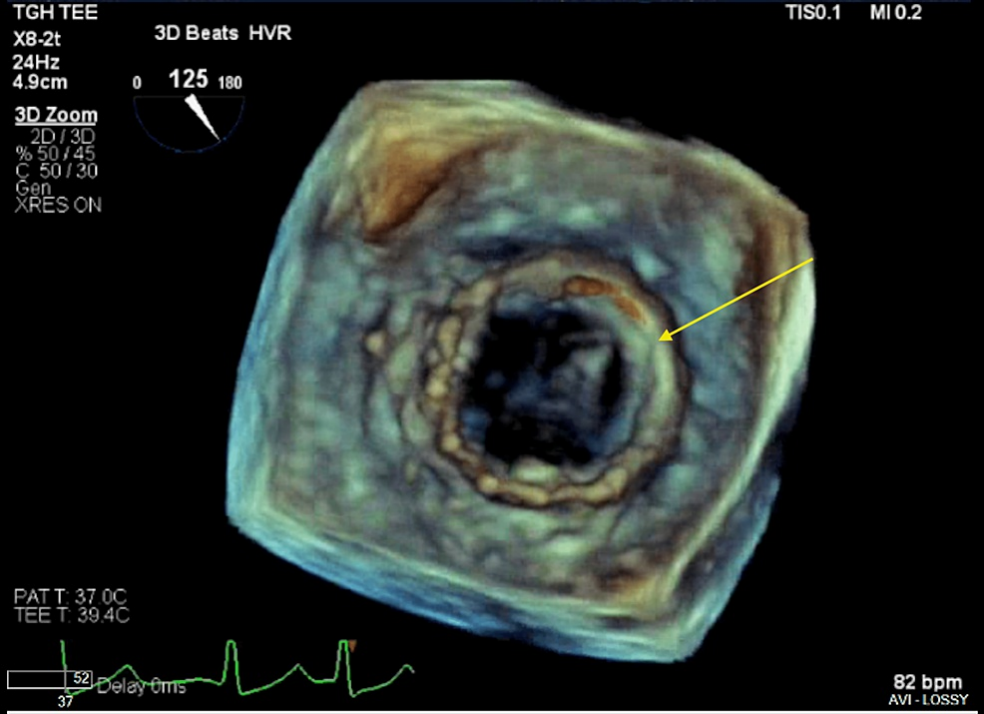
Three-dimensional TEE of the valve-in-valve transcatheter mitral valve replacement (yellow arrow) (surgeon’s view). TEE: Transesophageal Echocardiogram

**Figure 6:**
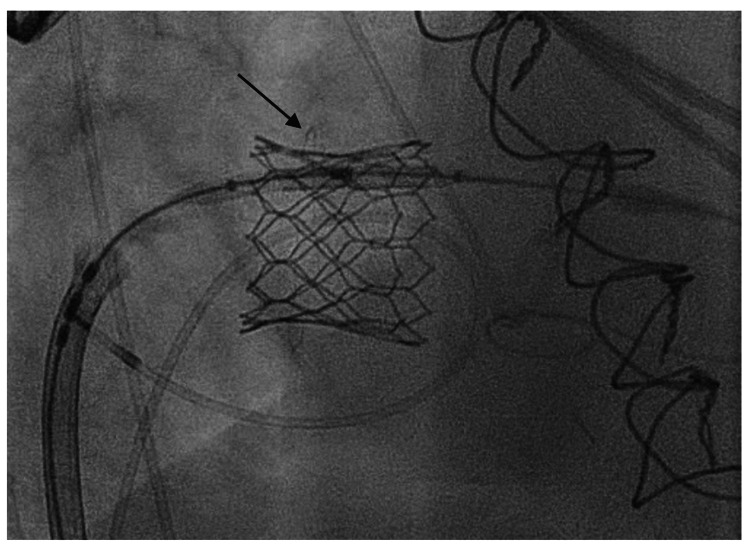
Fluoroscopy in the right anterior oblique (RAO) projection showing the newly deployed transcatheter mitral valve replacement inside the prior bioprosthetic valve (black arrow).

She underwent left and right heart catheterization with demonstrated normal coronary arteries, normal cardiac output, and index, and borderline post-capillary pulmonary hypertension. Despite no evidence of occult infection, due to the early bioprosthetic failure, the patient was evaluated by the Infectious Disease service to rule out prosthetic endocarditis. It was ruled out because there was no history of fevers, negative blood cultures, normal white blood cell counts, and no vegetation on echocardiography. The patient was subsequently assessed by the Structural Heart Team for intervention planning. The patient was deemed to be of prohibitive surgical risk (a Society of Thoracic Surgeons Short-Time Risk (STS) score of 13.79%). She was then referred for transcatheter mitral valve replacement. The patient underwent TMVR via a transfemoral approach using a 29mm Edwards SAPIENS Ultra valve, which was deployed inside the degenerative prior St. Jude Medical bioprosthesis valve (Figures 4-6).

The patient tolerated the procedure well, with an immediate improvement in her symptoms. There were no procedural complications. She was hospitalized for 11 days, of which 10 were intended for patient studies and one was post-procedure with subsequent discharge to home with self-care. At the follow-up one month later, the patient remained well with the resolution of her symptoms and returned to her normal daily activities.

## Discussion

Pibarot et al. presented a four-step algorithm for the detection, staging, and categorization of mitral bioprosthetic valve dysfunction and failure. Diagnosis of structural bioprosthetic valve dysfunction (BVD) after bioprosthetic mitral valve replacement requires the use of a transthoracic echocardiogram (TTE), transesophageal echocardiogram (TEE), or computerized tomography (CT). Other causes, such as thrombosis, endocarditis, and structural and nonstructural valve dysfunction, must be excluded. If the presence of structural valve dysfunction is imminent, staging and clinical consequences must be evaluated [4]. Based on this literature review, our patient was categorized as stage two structural bioprosthetic valve dysfunction with a mean transmitral gradient of 7 mmHg and criteria two for mitral valve reintervention by the presence of hemodynamic and symptomatic features.

Another study by Long et al. demonstrated that the transcatheter mitral valve replacement inside a prior bioprosthesis ("valve-in-valve") can be performed within a short procedure time, with minimal blood loss, and a very low risk of intraprocedural mortality, making it a viable option for dealing with potentially high-risk patients [5]. Belluschi et al. also demonstrated that although redo-surgery is the gold standard in the majority of bioprosthetic valve dysfunction (BVD) patients, the transcatheter procedure was found to be effective for the treatment of prosthetic valve deterioration, excluding cases of endocarditis or malpositioning. Nevertheless, each patient should have a tailored evaluation with the Heart Team, with strong consideration for surgical risk and potential anatomic issues [6].

## Conclusions

This case demonstrates a very premature bioprosthetic valve failure. Early failure should be suspected in patients presenting with clinical heart failure at any point during their post-implantation course. Surgical or minimally invasive approaches should be discussed and tailored to the patient’s clinical comorbidities and desires.

## References

[1] Hensey M, Brown RA, Lal S (2021). Transcatheter mitral valve replacement: an update on current techniques, technologies, and future directions. JACC Cardiovasc Interv.

[2] (2022). Mitral valve regurgitation. Mayo Clinic.

[3] Enta Y, Nakamura M (2021). Transcatheter mitral valve replacement. J Cardiol.

[4] Pibarot P, Herrmann HC, Wu C (2022). Standardized definitions for bioprosthetic valve dysfunction following aortic or mitral valve replacement: JACC state-of-the-art review. J Am Coll Cardiol.

[5] Long A, Mahoney P (2018). Transcatheter mitral valve-in-valve and valve-in-ring replacement in high-risk surgical patients: feasibility, safety, and longitudinal outcomes in a single-center experience. J Invasive Cardiol.

[6] Belluschi I, Buzzatti N, Castiglioni A, De Bonis M, Maisano F, Alfieri O (2021). Aortic and mitral bioprosthetic valve dysfunction: surgical or percutaneous solutions?. Eur Heart J Suppl.

